# Characterization and ex vivo functional properties of NKG2C^+^ NK cells with adaptive features in breast tumors

**DOI:** 10.3389/fimmu.2026.1995578

**Published:** 2026-10-27

**Authors:** María Belén Bordignon, Ayelén Ivana Pesce Viglietti, Luciana Sabatini, Natalia Elizabeth Nuñez, Mora Amat, Verónica Fabiano, Santiago Nelson Sánchez, Sebastián Aebi, Paola Gabriela Brunacci, Nicolas Flaibani, Azul Perazzolo, Marcelo Triveño Quiroz, Florencia Cappuccio, Darío Ricardo Martín, Carlos Martín Loza, Marianela Ruth Ramírez, Federico Coló, José Mordoh, Estrella Mariel Levy

**Affiliations:** 1 Centro de Investigaciones Oncológicas-Fundación Cáncer (FUCA), Ciudad Autónoma de Buenos Aires, Argentina; 2 Servicio de Cirugía Oncoginecológica y Mastología, Instituto Alexander Fleming (IAF), Ciudad Autónoma de Buenos Aires, Argentina; 3 Unidad de Mastología, Hospital Municipal “Profesor Dr. Bernardo Houssay”, Vicente López, Buenos Aires, Argentina; 4 Servicio de Anatomía Patológica, del Instituto Alexander Fleming (IAF), Ciudad Autónoma de Buenos Aires, Argentina; 5 Servicio de Anatomía Patológica, Hospital Municipal “Dr. Bernardo Houssay”, Vicente López, Buenos Aires, Argentina; 6 Laboratorio de Evolución, Instituto de Ecología, Genética y Evolución de Buenos Aires (IEGEBA), Facultad de Ciencias Exactas y Naturales - Universidad de Buenos Aires (FCEN-UBA), Ciudad Autónoma de Buenos Aires, Argentina

**Keywords:** adaptive natural killer cells, breast cancer, cancer immunotherapy, dendritic cells, tumor microenvironment

## Abstract

**Introduction:**

Adaptive Natural Killer cells are a specialized NK-cell subset with enhanced antibody-dependent effector functions, making them attractive candidates for cancer immunotherapy. Although circulating adaptive NK cells have demonstrated potent antitumor activity, their presence and functional status within the breast tumor microenvironment (TME) remain largely unknown.

**Methods:**

Here, we investigated the infiltration, phenotype, functional properties, and clinical relevance of NKG2C^+^ NK cells with adaptive features (aNK) in treatment-naïve breast cancer. Using paired peripheral blood, tumor, and adjacent non-tumoral breast tissue samples, together with functional assays and transcriptomic analyses from The Cancer Genome Atlas (TCGA), we characterized tumor-infiltrating aNK cells and evaluated their association with clinical outcome.

**Results:**

aNK cells are present within breast tumors while preserving their phenotype and enhanced effector functions. Tumor-infiltrating aNK cells retained robust antibody-dependent degranulation and IFN-γ production. Complementary studies using peripheral blood-derived aNK cells further demonstrated their superior capacity to promote dendritic cell maturation, supporting their immunoregulatory potential. Moreover, we analyzed an adaptive NK-associated transcriptional signature in TCGA breast cancer samples and found that higher signature scores were associated with a longer progression-free interval and an immune microenvironment enriched in specific CD8^+^ T-cell populations.

**Discussion:**

These findings indicate that aNK cells are functionally resilient to the suppressive breast TME, maintaining some specialized functions and immunoregulatory properties after tumor infiltration. Their persistence and association with favorable clinical outcome provide a rationale for further investigating their potential role in antibody-based immunotherapy and as a possible target for therapeutic strategies in BC.

## 1 Introduction

Natural Killer (NK) cells are key mediators of antitumor immunity, contributing not only to the direct elimination of transformed cells but also to the regulation of innate and adaptive immune responses, through cytokine production and crosstalk with dendritic cells (DCs), macrophages, and T lymphocytes (1). These properties have made NK cells attractive targets for cancer immunotherapy. However, their therapeutic potential is frequently limited by the tumor microenvironment (TME), which promotes NK-cell dysfunction through multiple mechanisms, including chronic exposure to inhibitory signals, metabolic stress, and suppressive cytokines. As a result, tumor-infiltrating NK cells often display impaired cytotoxicity, reduced cytokine production, and altered receptor expression (2–9). While these defects have been extensively documented for the overall NK-cell population, considerably less is known about how individual NK-cell subsets respond to the TME. Whether distinct NK-cell subsets exhibit differential susceptibility to these suppressive mechanisms remains poorly characterized, although adaptive NK cells have been shown to be intrinsically resistant to MDSC-mediated suppression (10).

Adaptive NK cells represent a highly specialized NK-cell subset that arises predominantly following human cytomegalovirus (HCMV) infection (11). Unlike conventional NK (cNK) cells, adaptive NK cells undergo stable transcriptional and epigenetic reprogramming that confers memory-like characteristics, enhanced longevity (12). Phenotypically, adaptive NK cells are characterized by the expression of NKG2C and CD57 together with reduced expression of FcϵRIγ, NKG2A, and PLZF and are characterized by enhanced antibody-dependent cellular cytotoxicity (ADCC) and increased production of inflammatory cytokines following IgG1 antibody-dependent activation. Collectively, these features make adaptive NK cells particularly attractive candidates for therapeutic strategies based on monoclonal antibodies (13–15).

Breast cancer (BC) represents a relevant clinical setting in which to investigate the biology of adaptive NK cells. Among BC therapies antibodies such as trastuzumab (TRZ) have significantly improved the outcome of patients with HER2-positive disease, and part of their clinical efficacy depends on NK-cell-mediated ADCC (16). We previously demonstrated that adaptive NK cells are present in the peripheral blood (PB) of BC patients, where they preserve their characteristic phenotype and display superior responses against trastuzumab-coated HER2-positive tumor cells compared with cNK cells (17). Furthermore, we showed that although neoadjuvant anti-HER2 therapy remodels the circulating NK-cell compartment, the adaptive subset is preserved and even expands following treatment (17). Together, these findings suggest that adaptive NK cells constitute a stable and highly functional NK-cell population during antibody-based therapy.

Whether these specialized properties are maintained after adaptive NK cells enter the tumor microenvironment remains unknown. Although several studies have described phenotypic and functional alterations in tumor-infiltrating NK cells, most have analyzed NK cells as a single population, overlooking the marked heterogeneity that exists within this lineage (18).

Very recently, using single-cell RNA-sequencing, Lozada et al. (2026) profiled NK cells across cancer and uncovered a dichotomous phenotypic and functional landscape of tumor-infiltrating NK cells (19). They found a tissue-resident adaptive subset exhibited exclusively pro-inflammatory *IFNG-*driven profiles and was associated with prolonged survival in both primary and metastatic tumor settings. However, it remains unclear whether adaptive NK cells infiltrate breast tumors, preserve the functional features that distinguish them from cNK cells, or contribute to shaping the local immune response. Answering these questions is essential to determine whether adaptive NK cells simply represent another NK-cell subset within the tumor or whether they retain specialized functions that could be therapeutically exploited.

Beyond their well-established cytotoxic activity, NK cells also contribute to the regulation of adaptive immune responses through reciprocal interactions with DCs. NK cells promote DC maturation and cytokine production, whereas mature DCs further enhance NK-cell activation, establishing a positive feedback circuit that supports the development of effective antitumor T-cell responses (20, 21). Given the enhanced functional properties of adaptive NK cells, it is conceivable that this specialized subset may also exhibit superior immunoregulatory capacity. However, whether adaptive NK cells differ from cNK cells in their ability to promote DC maturation has received little attention.

A central unanswered question is whether the specialized biological program that defines adaptive NK cells is preserved after exposure to the suppressive TME. Using paired PB, tumor, and adjacent non-tumoral breast tissue (NTB) samples together with functional and transcriptomic analyses, we investigated whether NKG2C+ NK cells with adaptive-associated characteristics (aNK) preserve their specialized phenotype and function after infiltrating human breast tumors. We show at cellular level that aNK cells are present within breast tumors while preserving their phenotypic and functional characteristics, evaluated by flow cytometry. Moreover, aNK cells retain superior antibody-dependent effector and immunoregulatory functions, promote DC maturation more efficiently than cNK cells, and are associated with favorable clinical outcomes and a T cell microenvironment enriched in antigen-experienced cells. Together, these findings identify aNK cells as a functionally resilient NK-cell subset capable of preserving specialized effector and immunoregulatory functions within the breast TME, providing a rationale for future therapeutic strategies aimed at harnessing this population to improve antibody-based cancer immunotherapy.

## 2 Material and methods

### 2.1 Tumor cell line

The human HER2-positive breast cancer cell line BT-474 (ATCC HTB-20) was maintained in DMEM/F12 medium supplemented with 10% fetal bovine serum (FBS; Natocor), 3.5 ng/mL sodium selenite, 0.1 mM ascorbic acid, 300 μg/mL galactose, 13.6 mM sodium pyruvate, 5 μg/mL recombinant human insulin, 0.29 mg/mL L-glutamine, 100 U/mL penicillin, and 10 μg/mL streptomycin at 37 °C in a humidified incubator containing 5% CO_2_. Cells were detached using PBS containing EDTA and immediately used for the indicated experiments.

### 2.2 Human samples collection and processing

Peripheral blood, primary breast tumor tissue, and paired adjacent non-tumoral breast tissue (NTB) were prospectively collected from HCMV seropositive systemic treatment-naïve BC patients undergoing surgical resection. Moreover, the cohort was restricted to patients with ≥15% NKG2C^+^ NK cells in peripheral blood. This cutoff was established according to the same criterion used in previous works (17, 22), corresponding to the mean percentage of NKG2C^+^ NK cells plus two standard deviations calculated in the HCMV-seronegative population. The study was approved by the Institutional Ethics Committee of Instituto Alexander Fleming (Version: 1.1, Date: 21 dec 2022) and the Central Ethics Committee of Buenos Aires Province (Version: 1.0, Date 1 feb 2024). Written informed consent was obtained from all participants before sample collection. Peripheral blood samples were collected on the day of surgery, prior to the procedure. Clinical characteristics of the study cohort are summarized in **Table 1**.

**Table 1:** Breast cancer patients included in the study.

Clinical features	Clasification	n
Age: average (range)		54 (32-82)
Stage	I	19 (37%)
	II	21 (41%)
	III	9 (18%)
	ND	2 (4%)
Grade	1	3 (5%)
	2	36 (71%)
	3	10 (20%)
	ND	2 (4%)
Histological type	Ductal	36 (71%)
	Lobular	9 (18%)
	Mixed	2 (4%)
	Other	4 (7%)
Subtype	Luminal A	22 (43%)
	Luminal B	22 (43%)
	HER2	3 (6%)
	TN	4 (8%)

### 2.3 Isolation of human peripheral blood mononuclear cells

PBMCs were isolated from patient blood samples by density-gradient centrifugation using Ficoll-Paque PLUS (GE Healthcare). PBMCs were used for phenotypic and functional analyses, employing 6 × 10^5^ and 1 × 10^6^ cells per assay, respectively.

### 2.4 Measurement of anti-human cytomegalovirus IgG antibody

Anti-CMV IgG titer was quantitatively determined in plasma samples at the Clinical Analysis Laboratory of Alexander Fleming Institute by enzyme-linked fluorescence assay technique, using the VIDAS CMV IgG kit (Biomerieux), according to the manufacturer’s specifications. The results were reported in arbitrary units/mL (AU/mL) as negative (< 4.0 AU/mL) or positive (≥ 4.0 AU/mL) without equivocal range.

### 2.5 Generation of monocyte-derived dendritic cells

Buffy coats from healthy HCMV-seropositive donors were obtained and PBMCs were isolated by Ficoll density-gradient centrifugation. Cells were cryopreserved in liquid nitrogen until use. After thawing monocytes were isolated from freshly prepared PBMCs obtained from healthy donor buffy coats by positive immunomagnetic selection using CD14 MicroBeads (Miltenyi Biotec) according to the manufacturer’s instructions. Cell purity (>90%) was confirmed by flow cytometry.

Purified monocytes were cultured at 1 × 10^6^ cells/mL in complete RPMI medium (RPMI-1640 supplemented with 10% FBS, 2 mM L-glutamine, 100 U/mL penicillin, and 100 μg/mL streptomycin) supplemented with recombinant human IL-4 (50 ng/mL; PeproTech) and GM-CSF (150 ng/mL; Laboratorio Pablo Casará, Buenos Aires, Argentina) for 5 days at 37 °C in a humidified atmosphere containing 5% CO_2_. Immature dendritic cells generated under these conditions were phenotypically confirmed by flow cytometry as CD14^−^CD11c^+^CD83^−^CD86^low^ prior to use in coculture experiments. On day 5, immature dendritic cells were harvested, washed once with complete RPMI medium, and immediately used for coculture experiments.

### 2.6 Isolation of aNK cells

Cryopreserved PBMCs were thawed, and NK cells were purified by negative immunomagnetic selection as described above. For FACS aNK cell isolation, viable cells were identified by staining with Fixable Viability Stain 510 (FVS510) (BD) in PBS, followed by surface staining with fluorochrome-conjugated antibodies against CD3 APC-H7, CD56 APC, and NKG2C BV421 for 30 min at 4 °C in RPMI 10% human serum albumin. After washing, cells were resuspended at 1 × 10^7^ cells/mL and kept on ice until sorting.

aNK (FVS510^−^CD3^−^CD56^+^NKG2C^+^) and cNK (FVS510^−^CD3^−^CD56^+^NKG2C^−^) cell populations were sorted using a BD FACSAria Fusion cell sorter. Sorted cells (>95% purity) were collected in RPMI-1640 containing 50% FBS, washed, and allowed to recover overnight in complete RPMI medium at a density of 1–2 × 10^6^ cells/mL in the presence of low-dose IL-15 (0.3 ng/mL).

### 2.7 Tumor tissue processing

Fresh breast tumor specimens and NTB were processed within 2 h after surgical resection. Tumor and NTB samples were identified and selected based on macroscopic examination by a pathologist. Therefore, NTB tissue may have contained areas of inflammation or micrometastases. Tissues were cut in 1–2 mm^3^ fragments and then mechanically and enzymatically dissociated in RPMI-1640 medium supplemented with collagenase IV (1 mg/mL; Sigma-Aldrich), DNAse I (0.1 mg/mL; Roche), hyaluronidase (0.1 mg/mL; Sigma-Aldrich), and penicillin/streptomycin using C Tubes (Miltenyi Biotec) and a gentleMACS Dissociator (Miltenyi Biotec). Two sequential rounds of mechanical and enzymatic dissociation were performed, each followed by incubation for 30 min at 37 °C.

Enzymatic digestion was stopped by adding complete RPMI medium. Cell suspensions were filtered through 70-μm cell strainers, washed with 0.9% NaCl, and immediately processed for flow cytometric analyses or functional analyses.

### 2.8 Flow cytometry

PBMCs and tumor-derived single-cell suspensions were stained for flow cytometric analysis of NK-cell subsets and other immune cell populations. Dead cells were excluded using FVS510. Cells were incubated with fluorochrome-conjugated antibodies (**Supplementary Table 1**) for 30 min at 4 °C in the dark, washed, and resuspended in PBS containing 2% FBS before acquisition.

For intracellular staining, cells were fixed and permeabilized using the Cytofix/Cytoperm™ Fixation/Permeabilization Kit (BD Biosciences) according to the manufacturer’s instructions before incubation with intracellular antibodies.

Samples were acquired on a FACSCanto II flow cytometer (BD Biosciences), and data were analyzed using FlowJo v10 software (Tree Star, USA).

### 2.9 Functional assays

#### 2.9.1 PMA/ionomycin stimulation

PBMCs and tumor-derived single-cell suspensions were stimulated with phorbol 12-myristate 13-acetate (PMA; 50 ng/mL) and ionomycin (1 μg/mL) for 3 h at 37 °C in the presence of CD107a PE-Cy7 antibody. Monensin (GolgiStop; BD Biosciences) was added after the first hour of stimulation to assess intracellular cytokine production. After stimulation, cells were stained for surface markers, fixed, permeabilized, and incubated with antibodies against intracellular proteins prior to flow cytometric analysis.

#### 2.9.2 CD16-mediated activation of intratumoral NK cells

Forty-eight-well plates were coated with the agonistic anti-CD16 antibody 3G8 (BioLegend; 5 µg/mL in PBS) for 2 h at 37 °C. After removing the coating solution, single-cell suspensions obtained from breast tumors or PBMCs were seeded in complete RPMI medium supplemented with IL-15 (1 ng/mL). Fluorochrome-conjugated anti-CD107a antibody was added at the beginning of the culture to monitor degranulation, and monensin was added after 1 h. Cells were incubated 16 hs at 37 °C. The following day, surface and intracellular staining were performed as described above, and samples were analyzed by flow cytometry.

### 2.10 Dendritic cell maturation and NK cell activation assay

Immature monocyte-derived DCs were cocultured with purified NK, aNK, or cNK cells from the PBMCs of five healthy donors, whose peripheral blood aNK-cell frequencies ranged from 25% to 55.9%, and TRZ-opsonized BT-474 cells. BT-474 cells were opsonized with TRZ for 20 min at room temperature at a final concentration of 10 µg/mL (23). Cells were cocultured at a 1:1:1 ratio (DC: NK) in complete RPMI medium at a final density of 3 × 10^5^ cells/mL in 48-well plates. After 24 h of coculture, cells were harvested to evaluate both DC maturation and NK cell activation phenotypes by flow cytometry. For DC maturation, cells were stained with antibodies against CD11c, CD80, CD83, and CD86. Immature DCs cultured alone served as the negative control, whereas DCs stimulated with a standard maturation cocktail—consisting of IL-1β (10 ng/mL), TNF-α (20 ng/mL), IL-6 (50 ng/mL), and prostaglandin E_2_ (PGE2; 1 µg/mL)—served as the positive control.

To assess NK cell activation, harvested cells were stained with anti-CD25, anti-CD69, anti-CD137, and anti-HLA-II antibodies. Baseline marker expression was established by culturing NK cells alone under identical conditions. Because aNK and cNK cell subsets had been pre-isolated by fluorescence-activated cell sorting (FACS) and retained their labeling for at least 48 h, additional lineage selection antibodies were omitted during staining.

### 2.11 Statistical analysis

To assess the clinical significance of aNK-cell infiltration, transcriptomic data from The Cancer Genome Atlas (TCGA) breast invasive carcinoma (BRCA) cohort were analyzed using the Gene Expression Profiling Interactive Analysis (GEPIA3) platform (24, 25). The score was calculated using the GEPIA3 expression analysis framework, in which expression-based signatures are computed as the first principal component (PC1) derived from the expression levels of the input genes. Kaplan–Meier survival analyses were performed using the breast invasive carcinoma (BRCA) cohort (n = 1,070) to evaluate the association between aNK cell associated transcriptional signature and overall survival (OS) and Progression-free interval (PFI).

aNK-cell infiltration was estimated using a 15-gene signature generated from the scRNA-seq associated to aNK cells (GZMH, KLRG1, S1PR5, FCRL6, CD8A, KLRK1, KLRC4, TTC16, FGFBP2, FNDC9, NKG7, KIR2DL3, KLRC3, PTGDR, KLRC2 (19). Patients were stratified into high- and low-aNK infiltration groups according to the median expression score of the aNK gene signature. Survival curves were compared using the log-rank (Mantel–Cox) test, and hazard ratios (HRs) were estimated using the Cox proportional hazards model implemented in GEPIA3 (25).

Correlation analyses were also performed using GEPIA3. Pearson correlation coefficients were calculated to evaluate the association between the aNK-associated transcriptional signature and a T-cell exhaustion associated signature composed of HAVCR2, TIGIT, LAG3, PDCD1, CXCL13, and LAYN.

GraphPad Prism 9.0.1 was used for graphs and analysis. First, we evaluated the normality of the residuals of the data with the Shapiro–Wilk normality test and the qq-plot. Then, we compared samples using *t* test and, or Mann–Whitney tests. One way ANOVA, mixed model analysis (with Restricted Maximum Likelihood method) (or non-parametric Friedman test) with Dunns post-tests and two-way ANOVA with Sidak’s post-tests was used for multiple comparison analysis with NK subpopulation or/and tissue as a fixed effect and patient as a random effect.

For DC maturation experiments, we fitted generalized linear mixed models (GLMMs), with Treatment as a fixed effect and Donor as a random effect. Treatment was defined by the combination of NK-cell subset and BT474/TRZ condition. DC maturation was assessed using the markers CD83, CD80, and CD86. For NK-cell activation experiments, we similarly fitted GLMMs with Treatment as a fixed effect and Donor as a random effect, with Treatment defined by the combination of NK-cell subset and BT474/TRZ condition. NK-cell activation was assessed using CD25, CD69, CD16, CD137, and HLA-II markers.

For CD25, CD69, CD16, CD137, HLA-II, and CD83, we used a binomial distribution with a logit link and included an observation-level random effect to account for overdispersion. For CD80 and CD86 mean fluorescence intensity (MFI), we used negative binomial distributions with log links. For the DC maturation markers (CD83, CD80, and CD86), we also performed analyses stratified by NK-cell subset. In these analyses, comparisons were restricted to the following *a priori* contrasts NK_DC_BT474_TRZ versus NK_DC_BT474 and NK_DC_BT474_TRZ versus DC_BT474_TRZ.

All comparisons between treatments were defined *a priori* and evaluated using contrasts derived from the fitted models. Inference was based on model-derived contrasts, with estimated effect sizes, corresponding 95% confidence intervals, and Wald tests reported. All models were fitted using the glmmTMB package (26) in R, and model diagnostics were assessed using the DHARMa package (27). Because the comparisons were based on the same donor samples, p-values were adjusted for multiple testing using the false discovery rate (FDR) approach with the Benjamini–Hochberg procedure (**Supplementary File**).

## 3 Results

### 3.1 NKG2C^+^ NK cells with adaptive-associated characteristics infiltrate human breast tumors while retaining their hallmark features

Adaptive NK cells have been extensively characterized in PB, lymph nodes, and other tissues; however, whether they infiltrate solid tumors while preserving their specialized phenotype remains largely unknown. To determine whether aNK cells can infiltrate breast tumors, paired PB, primary tumor, and NTB samples were collected from patients with previously untreated BC at the time of surgery (**Table 1**).

Single-cell suspensions obtained from PB, tumors and paired NTB were analyzed by multiparametric flow cytometry. Following exclusion of dead cells and selection of CD45^+^ and lymphocytes, NK cells were identified as CD3^−^CD56^+^ cells, and adaptive-like and conventional subsets were distinguished according to NKG2C expression (**Supplementary Figure 1**).

Consistent with previous reports describing the breast tumor immune microenvironment, the proportion of NK cells within the lymphocyte compartment was significantly lower in tumors than in PB or NTB (28). In contrast, breast tumors showed a trend toward enrichment of the CD56^bright^ NK-cell subset, which is typically associated with an immature, cytokine-producing phenotype, whereas the proportion of CD56^dim^ NK cells remained unchanged across the three compartments (**Figures 1A–C**).

**Figure 1:**
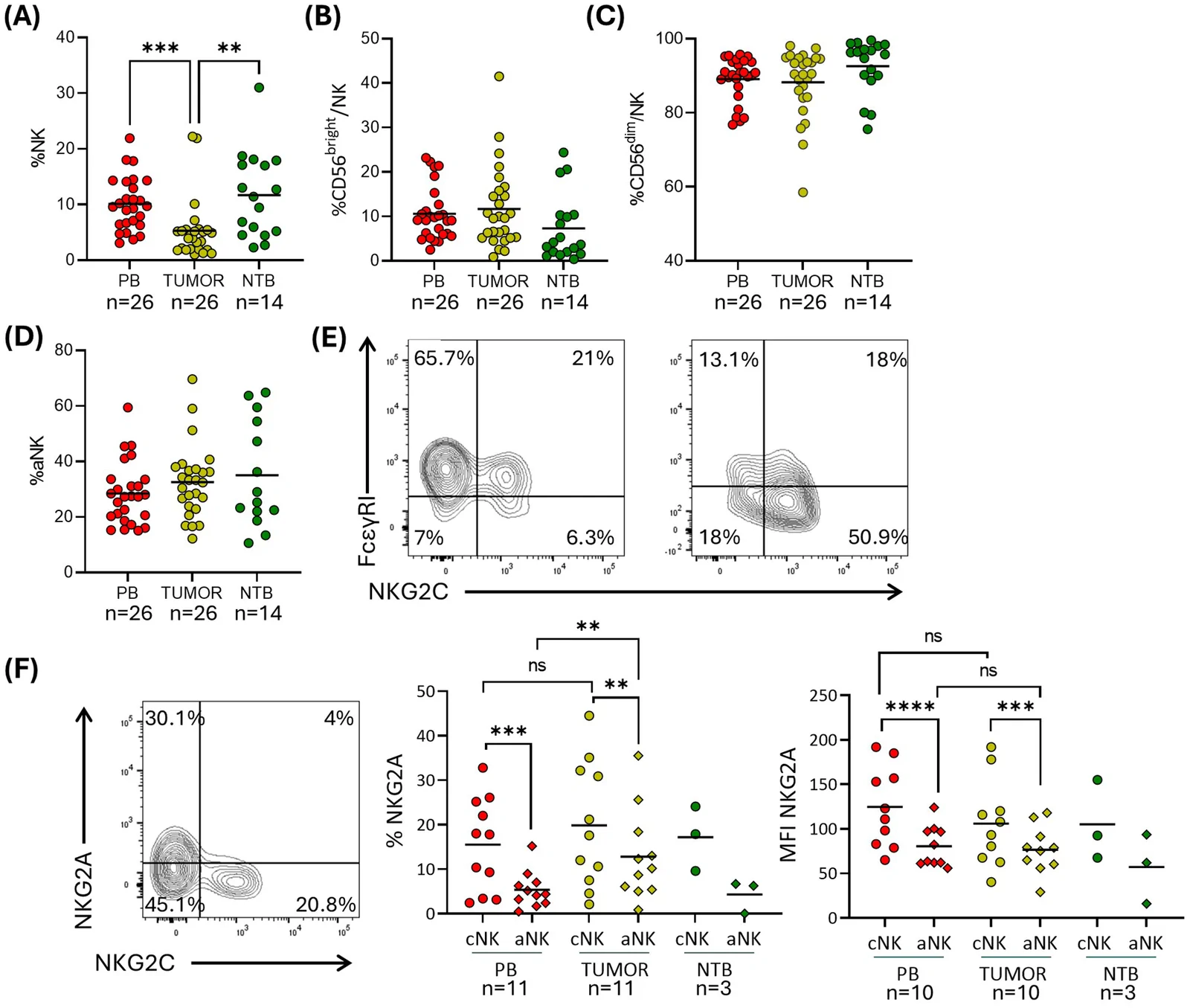
Proportion of NK cells **(A)**, CD56bright **(B)** and CD56dim **(C)** in PB, tumor and NTB. **(D)** Proportion of aNK cells (NKG2C+) in PB, tumor, and NTB. **(E)** Examples of expression of NKG2C and FcϵRIγ within NK cells in tumor tissue from two patients with BC. **(F)** Example of expression of NKG2C and NKG2A within NK cells in tumor tissue (left) and quantification of the percentage of expresión (center) and MFI (right) of NKG2A in aNK cells. Mixed-effects model (REML) with Tukey post test. ** p<0.01, *** p<0.001, ****=p<0.0001; ns, non significant.

Having established the overall distribution of NK cells across the different tissue compartments, we next examined whether the aNK cell subset was also represented within the tumor infiltrate. All 51 breast cancer patients included in the study were HCMV-seropositive and had an established peripheral aNK cell subset, defined as ≥15% of PB NK cells. Under these conditions, the frequency of aNK cells was remarkably similar in PB, tumor tissue, and NTB, with no significant differences among compartments (**Figure 1D**).

To verify that tumor-infiltrating aNK cells retained the canonical adaptive markers, first the expression of FcϵRIγ and NKG2A proteins was examined in representative samples. Tumor-infiltrating aNK cells exhibited some variability in FcϵRIγ. NKG2A expression remained characteristically low in aNK cells as previously associated with adaptive NK cells (29) but increased in tumor-infiltrating NK cells, indicating that the TME modulates selected phenotypic features without completely abolishing the aNK-cell program (**Figures 1E, F**).

Together, these findings demonstrate that aNK cells are present in human breast tumors while preserving features that distinguish this specialized NK-cell subset from cNK cells. Importantly, these observations indicate that infiltration of the breast TME does not erase the adaptive phenotype features that define aNK cells.

### 3.2 Adaptive NK-cell associated gene signature is linked with favorable clinical outcome in BC

Having demonstrated that aNK cells infiltrate human breast tumors while preserving their phenotypic identity, we next investigated whether their presence was associated with clinical outcome. We analyzed the TCGA breast cancer cohort using GEPIA3 and an adaptive NK-associated gene signature as a surrogate of intratumoral infiltration.

Kaplan–Meier analysis showed that patients with high adaptive NK-associated signature scores exhibited significantly longer progression-free interval (PFI) and overall survival (OS), than those with low scores (**Figure 2**). However, although the OS was statistically significant, the median between the two groups only differs in one month then might not be biologically relevant. The corresponding hazard ratios (HR) and median survival values are summarized in **Table 2**. These observations provide independent clinical support for the biological relevance of adaptive NK cells in human BC.

**Figure 2:**
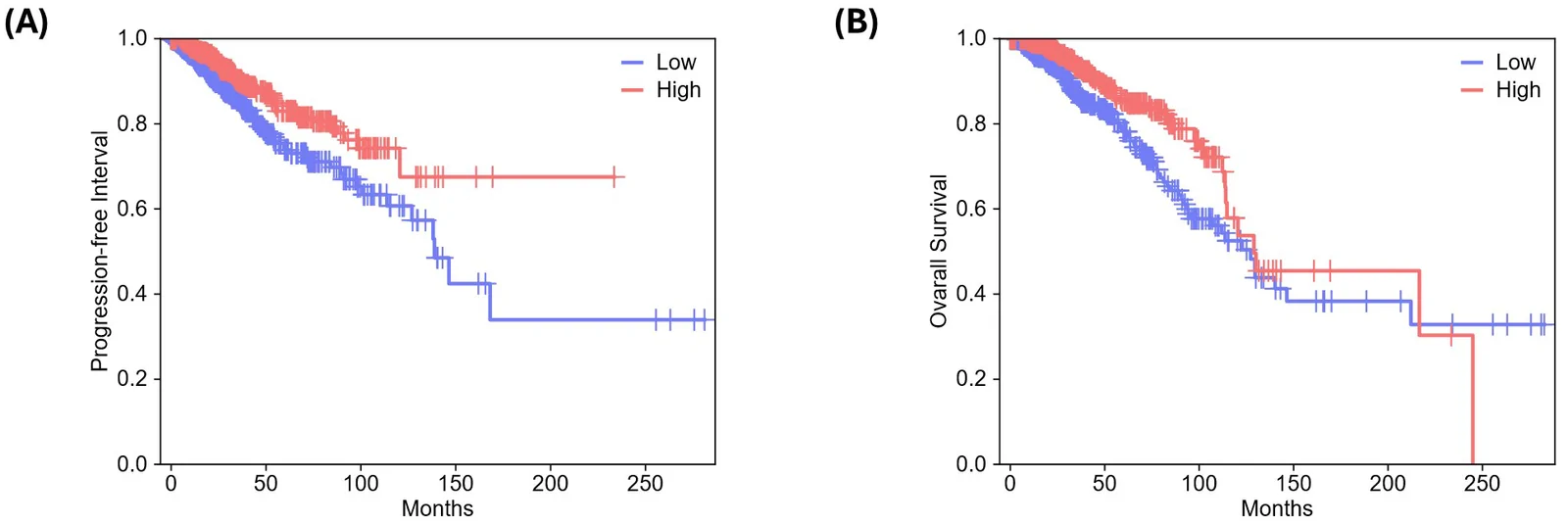
Kaplan-Meier plot for progression-free interval (PFI, **A**) and overall survival (OS, **B**) categorizing patients according to a high (red) or low (blue) score for the aNK cell genetic signature (n=1070).

**Table 2:** Statistical data for Kaplan-Meier analysis.

Clinical features	Median (high) n=535	Median (low) n=535	p-value	HR (95% IC)	p-value (HR)
PFI	inf	138.78	0.00194	0.59 (0.42-0.83)	0.00218
OS	128.99	127.24	0.00223	0.6 (0.43-0.84)	0.00249

### 3.3 Tumor-infiltrating aNK cells retain their adaptive-associated phenotypic features

Given the association between aNK-cell infiltration and favorable clinical outcome, we next investigated whether aNK cells retain their characteristic phenotype after entering the TME. Following tissue dissociation, the expression of representative activating and inhibitory receptors was analyzed by flow cytometry using both the percentage of positive cells and the mean fluorescence intensity (MFI). The expression of the various NK cell markers studied was quite heterogeneous and variable across the different tissues (**Figure 3**). As previously reported for circulating aNK cells (17), CD16 expression was lower in aNK than in cNK cells. Notably, CD16 expression was further reduced after tumor infiltration. Both adaptive and conventional NK-cell subsets exhibited an approximately 56% reduction relative to their circulating counterparts (**Figure 3A**).A comparable pattern was observed for NKp46 expression, although this effect was more pronounced in NTB when the proportion of positive cells was analyzed (**Figure 3B**).

**Figure 3:**
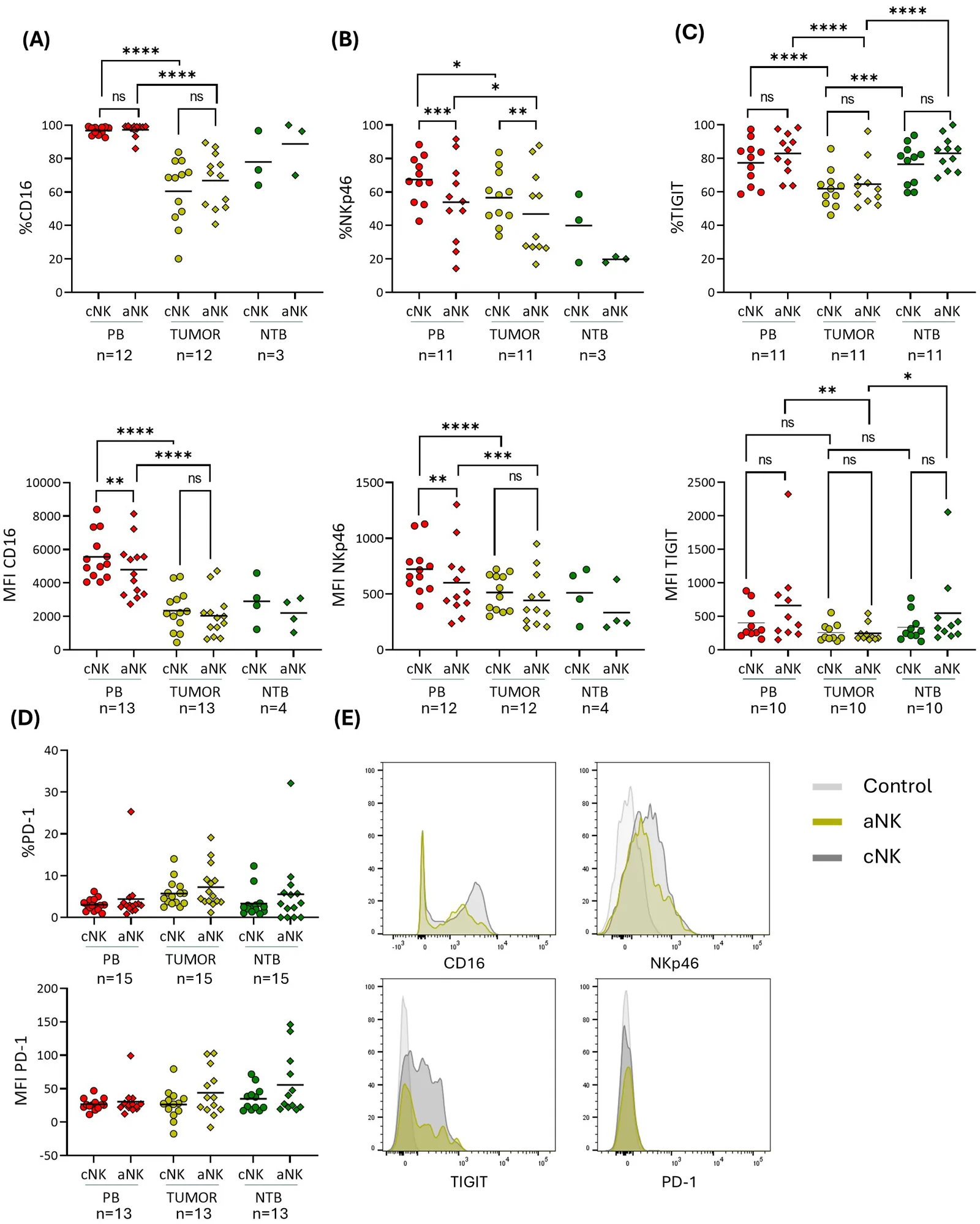
Percentage (top) and MFI (bottom) of aNK and cNK cells for surface receptors CD16 **(A)** and NKp46 **(B)** in PB, tumor and adjacent NTB. Percentage (top) and MFI (bottom) of aNK and cNK cells for TIGIT **(C)** and PD-1 **(D)** surface receptors in PB, tumor and NTB.). **(E)** Examples of expression of CD16, NKp46, PD-1 and TIGIT within aNK and cNK cells in breast tumor tissue. Two-way ANOVA with Sidak post-test. For CD16, NKp46 and NKG2A NTB samples were excluded from the statistical analysis. * p<0.05, ** p<0.01, *** p<0.001, *** p<0.001; ns, non significant.

To determine whether additional inhibitory pathways were affected, we next evaluated the expression of TIGIT and PD-1. Whereas TIGIT expression decreased within the tumor infiltrate, PD-1 showed a tendency to increase, particularly among aNK cells (**Figures 3C–E**).

Together, these findings indicate that although the breast TME induces selective phenotypic remodeling of both adaptive and conventional NK cells, aNK cells retain the selected adaptive-associated phenotypic features that distinguish this specialized lineage.

### 3.4 Tumor infiltrated aNK cells retain ex vivo superior effector functions

The association between aNK-cell infiltration and improved clinical outcome prompted us to determine whether this subset retains functional properties that could explain its favorable prognostic impact. Freshly isolated PBMCs and tumor and NTB derived single-cell suspensions were stimulated with PMA and ionomycin to assess the intrinsic functional capacity after strong pharmacological stimulation of adaptive and conventional NK cells independently of receptor-mediated activation. As previously described for circulating NK cells, adaptive NK cells displayed greater degranulation and produced higher levels of IFN-γ than cNK cells (17). Within the TME, however, this functional profile was only partially preserved. A higher proportion of tumor-infiltrating aNK cells produced significantly more IFN-γ than infiltrating cNK cells, whereas no significant differences in degranulation were observed between the two subpopulations. Notably, IFN-γ production by tumor-infiltrating aNK cells was comparable to that of circulating aNK cells (**Figures 4A, B**). These findings support that breast tumor infiltration does not impair the intrinsic cytokine-producing capacity that characterizes aNK cells.

**Figure 4:**
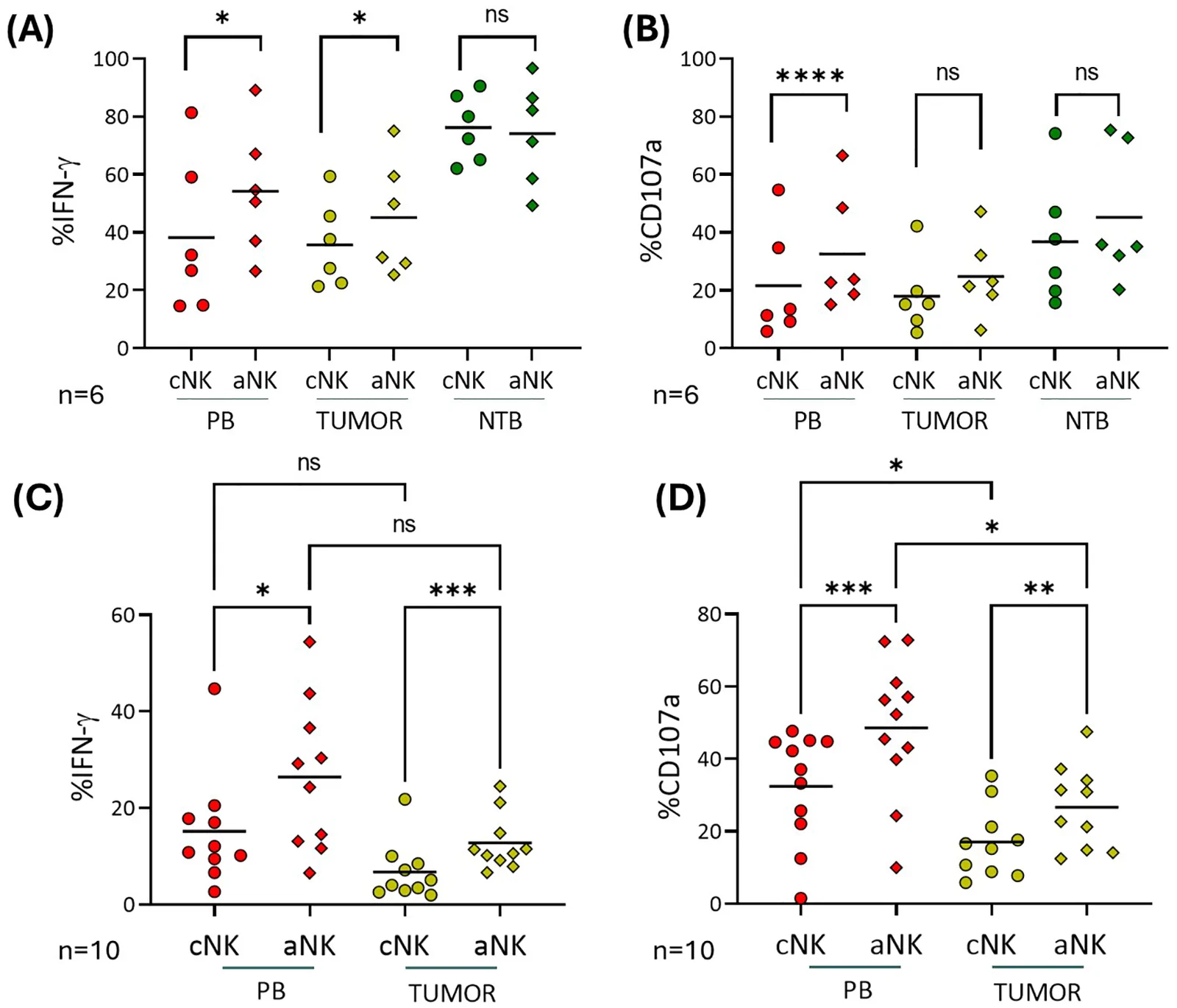
Percentage of aNK and cNK cells positive for IFN-γ **(A)** and CD107a **(B)** in PB, tumor and NTB after being stimulated for 3 hours with PMA and ionomycin. Percentage of aNK and cNK cells positive for IFN-γ **(C)** and CD107a **(D)** in PB and tumor after being stimulated for 16 hours with the anti-CD16 agonist antibody. Two-way ANOVA with Sidak post test.

Given the central role of CD16 signaling in aNK-cell responses, we next investigated whether the reduction in CD16 expression observed after tumor infiltration translated into impaired receptor function. Cells from breast tumors and PBMCs were stimulated with plate-bound agonistic anti-CD16 antibody (3G8) (**Supplementary Figure 2**). Despite the partial downregulation of CD16, aNK cells remained significantly more responsive than cNK cells, exhibiting higher IFN-γ production and enhanced degranulation. Overall degranulation was reduced in tumor-derived NK cells relative to PB. Nevertheless, aNK cells consistently outperformed their conventional counterparts following CD16 engagement (**Figures 4C, D**, controls are shown in **Supplementary Figure 3**). Together, these findings demonstrate that aNK cells retain their enhanced effector competence despite phenotypic remodeling and prolonged exposure to the immunosuppressive breast tumor microenvironment.

### 3.5 aNK cells promote more efficient DC maturation than conventional NK cells

Considering the superior functional capacity of aNK cells, we next investigated whether this subset differentially promotes DC maturation, using the maturation cocktail as a positive control (**Supplementary Figure 4A**). An autologous *in vitro* co-culture system was established using monocyte-derived immature DCs and either total NK cells or FACS-sorted aNK and cNK subsets obtained from the same HCMV-seropositive healthy donor (**Figure 5A**). Cells were cultured in the presence of HER2^+^ BT-474 breast cancer cells, either untreated or opsonized with TRZ. As expected, the addition of total NK cells enhanced DC maturation in response to TRZ-opsonized tumor cells, as evidenced by increased CD80 and CD86 expression and a higher proportion of CD83^+^ DCs (**Supplementary Figure 4B**). These results indicate that NK-cell activation during antibody-dependent tumor recognition promotes the acquisition of a mature DC phenotype.

**Figure 5:**
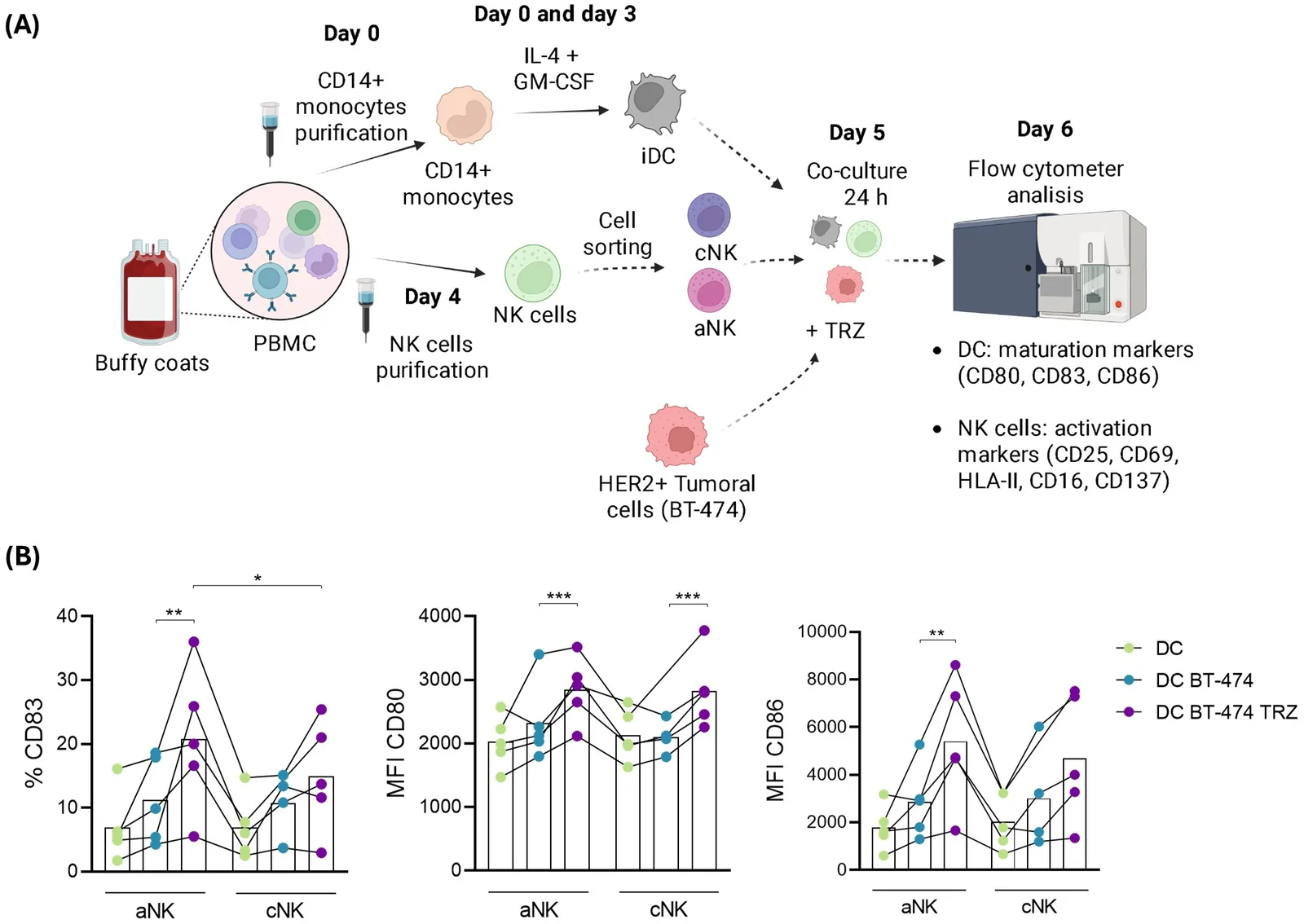
**(A)**. Schematic representation of the experimental workflow. NK cells were isolated from PBMCs of healthy donors by magnetic separation and subsequently sorted into aNK and cNK subsets based on NKG2C expression by fluorescence-activated cell sorting (FACS). Purified NK-cell subsets were then co-cultured with immature monocyte-derived DCs in the presence or absence of BT474 cells, either untreated or opsonized with trastuzumab (TRZ). **(B)** Expression of DCs maturation markers, CD83 (left), CD80 (middle) and CD86 (right), after co-culture with BT-474 cells plus TRZ, modulated by aNK or cNK cells (n=5) Wald-test. Created with BioRender.com.

We next asked whether aNK and cNK cell subsets differed in their ability to induce DC maturation. Although both subsets increased CD80 expression, only aNK cells significantly enhanced CD83 and CD86 expression in the presence of TRZ. Moreover, aNK cells induced significantly higher CD83 expression than cNK cells under identical experimental conditions (**Figure 5B**).

Collectively, these findings demonstrate that aNK cells are more efficient than cNK cells at promoting DC maturation following antibody-dependent activation. This enhanced capacity is consistent with the superior functional properties of aNK cells and identifies an additional immunoregulatory feature of this subset. Whether a similar interaction occurs within the breast TME remains to be determined.

To further characterize how DCs influence NK-cell activation during antibody-dependent responses, we evaluated the activation phenotype of aNK and cNK cells following co-culture. As expected, engagement of CD16 by antibody-opsonized tumor cells induced receptor internalization, which was further enhanced in the presence of DCs. Although CD16 surface expression decreased in both subsets, the magnitude of receptor downregulation was more pronounced in cNK cells than in aNK cells, indicating differential regulation of CD16 following antibody-dependent activation.

We next examined additional activation-associated molecules to determine whether aNK and cNK cells responded differently to DC interaction. The presence of DCs preferentially enhanced CD25 expression in cNK cells. In contrast, CD69 and HLA-II were similarly upregulated in both subsets after co-culture, consistent with reciprocal activation during NK–DC crosstalk (**Supplementary Figure 5**).

### 3.6 aNK-cell infiltration is associated with immune checkpoint-receptor-expressing CD8^+^ T cells

Given the effector and immunoregulatory functions of aNK cells, we next investigated whether their presence was associated with changes in the intratumoral CD8^+^ T-cell compartment. Breast tumors contained a higher proportion of CD8^+^ T lymphocytes than PB and exhibited increased expression of the PD-1 or TIGIT, consistent with chronic antigen stimulation within the TME (**Figures 6A–D**).

**Figure 6:**
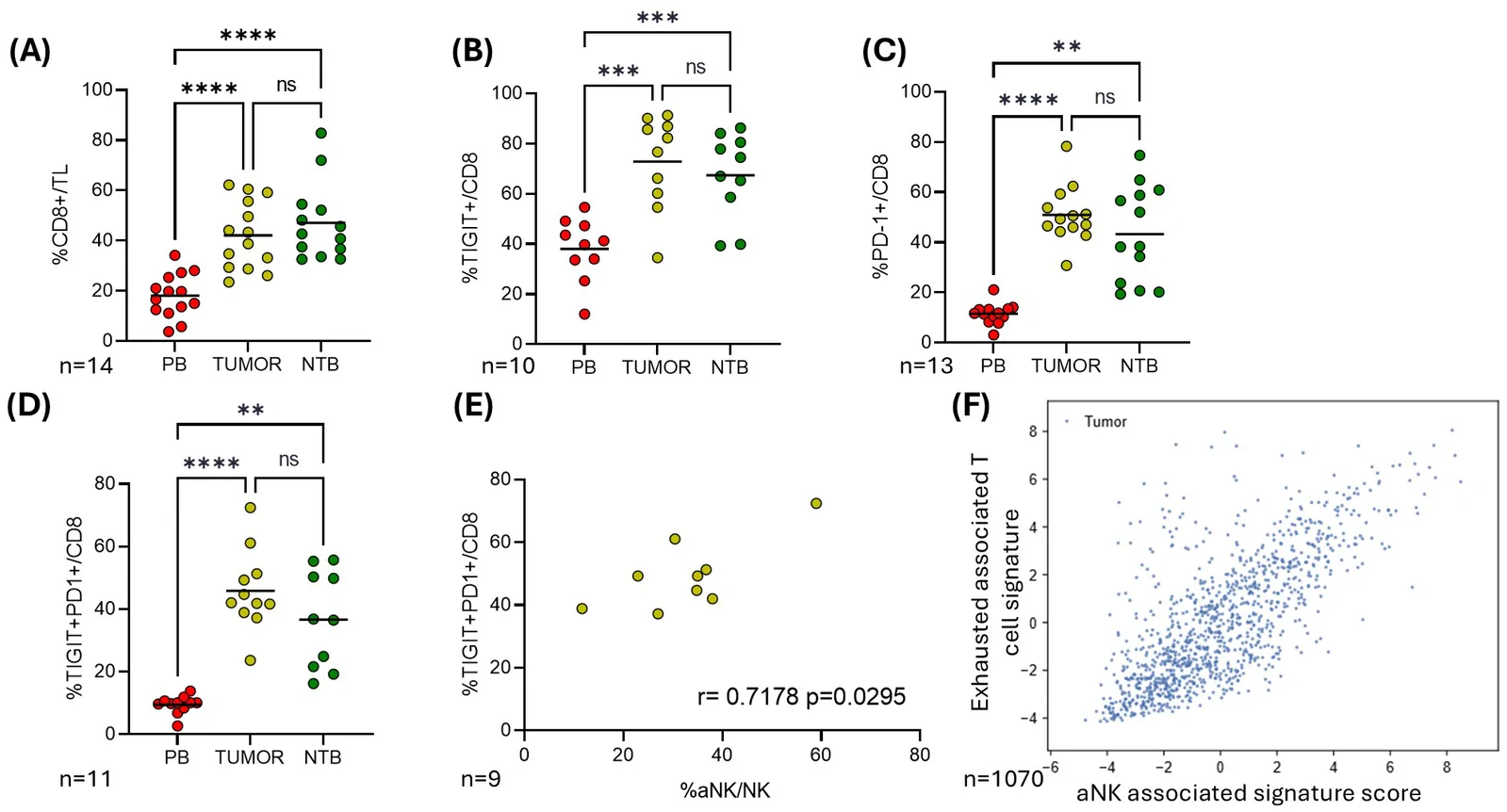
Proportion of CD8+ T lymphocytes **(A)**, CD8+TIGIT+ T lymphocytes **(B)** and CD8+ PD-1+ T lymphocytes **(C)** in peripheral blood (PB), tumor and adjacent non-tumor breast tissue (NTB). Proportion of CD8+ TIGIT+ PD-1+ T lymphocytes in peripheral blood (PB), tumor, and adjacent non-tumor breast tissue (NBT) **(D)**. One way ANOVA with Dunns post test. Pearson correlation of the proportion of aNK cells and CD8+ TIGIT+ PD-1+ T lymphocytes **(E)**. Pearson correlation between the genetic signature of aNK cells and exhausted associated CD8 T lymphocytes using TCGA breast cancer samples as a database and the GEPIA3 tool r= 0.73 p-value=1.42e-183 **(F)**. p<0.05, ** p<0.01, *** p<0.001, *** p<0.001; ns, non significant.

We then examined whether aNK-cell infiltration was associated with specific CD8^+^ T-cell phenotypes. No significant association was observed with either the frequency of total or PD1+CD8+ or TIGIT+CD8+ cells (**Supplementary Figure 6**). In contrast, the proportion of CD8^+^PD-1^+^TIGIT^+^ T cells positively correlated with aNK cells, suggesting that tumors enriched in aNK cells also harbor larger populations of antigen-experienced or chronically stimulated CD8^+^ T lymphocytes (**Figure 6E**).

To determine whether this association extended to an independent cohort, we analyzed transcriptomic data from the TCGA BC dataset using aNK-cell associated and exhausted associated CD8^+^ T-cell gene signatures. A significant positive correlation was observed between both signatures, independently supporting the association between aNK-cell infiltration and an immune microenvironment enriched in transcriptional programs associated with CD8^+^ T-cell exhaustion (**Figure 6F**).

## 4 Discussion

In this study, we demonstrate that aNK cells infiltrate human breast tumors while preserving some phenotypic and functional features that distinguish this subset from cNK cells. Furthermore, our findings indicate that aNK cells exhibit enhanced immunomodulatory properties, including an increased capacity to promote DC maturation, and that their presence within the TME is associated with improved clinical outcome. Together, these observations expand the current understanding of aNK-cell biology beyond the periphery and support the concept that aNK cells constitute a specialized NK-cell subset that remains functionally active within human breast tumors and is associated with a favorable immune contexture.

Adaptive NK cells have been extensively characterized in PB in the context of HCMV (30), whereas their role within solid tumors has only lately begun to emerge. Recent studies have identified adaptive NK cell populations across multiple human tumors and have shown that these cells display pro-inflammatory transcriptional programs associated with favorable clinical outcomes (19). Also, Sun et al. (2025) demonstrated that tumor-infiltrating adaptive NK cells in ovarian cancer exhibit tumor-specific immune memory, conferred by the interaction of adaptive NK cells and DC cells, along with enhanced cytotoxicity against autologous tumor cells and resistance to tumor-induced immunosuppression (31). Together, these studies support the concept that adaptive NK cells represent a specialized NK-cell subset with considerable therapeutic potential in solid tumors. Our study extends these observations by providing direct phenotypic and functional characterization of aNK cells in human BC, demonstrating that they could preserve enhanced CD16-dependent effector functions and immunoregulatory activity after tumor infiltration and that their presence is associated with improved clinical outcome and immunologically active TME.

An important strength of the present study lies in the experimental approach used to characterize aNK cells. Most current evidence regarding adaptive NK cells in solid tumors has been derived from transcriptomic analyses, including bulk and single-cell RNA sequencing datasets (19, 31), which infer the presence of adaptive NK-cell programs based on gene-expression signatures. While these approaches have considerably advanced our understanding of NK-cell heterogeneity, they provide limited information regarding protein expression and functional competence. In contrast, our study directly studied immune cells from freshly resected human breast tumors, allowing the phenotypic and functional characterization of aNK cells at the single-cell level. This approach enabled us not only to confirm their presence within the TME but also to demonstrate that they retain some specialized functional properties that cannot be inferred from transcriptomic analyses alone.

In our study, tumor-infiltrating aNK cells displayed phenotypic changes that were shared with cNK cells, including reduced expression of CD16 and NKp46 and increased expression of NKG2A, indicating that both NK-cell subsets are shaped by the local TME. Nevertheless, these changes did not abolish the characteristic adaptive phenotype. A methodological consideration is that tissue-derived and PB samples require different processing procedures. Although tumor and adjacent NTB tissues were processed in parallel using the same dissociation protocol, enzymatic digestion may affect the integrity or surface expression of some receptors. Rather, aNK cells maintained the adaptive-associated features that distinguish them from cNK cells, suggesting that environmental cues induce phenotypic adaptation without completely overriding their differentiation state. This observation is consistent with the remarkable stability that has been attributed to aNK cells following HCMV-driven expansion and supports the concept that lineage-specific programs are preserved (32) despite local environmental modulation.

One of the most striking findings of this study is that aNK cells retained ex vivo superior functional activity after infiltrating breast tumors. Despite the reduction in CD16 expression observed in both circulating and tumor-infiltrating cells, aNK cells remained more responsive than cNK cells following CD16-mediated stimulation and continued to produce higher levels of IFN-γ. Likewise, their intrinsic capacity to respond to PMA and ionomycin was preserved within the TME however these experimental settings have the limitation that they do not recapitulate physiological tumor recognition. These findings indicate that phenotypic adaptation does not necessarily translate into functional impairment and suggest that aNK cells preserve specific effector functions even under conditions that are generally considered suppressive for NK-cell activity (33, 34). The enhanced responsiveness of aNK cells following CD16 engagement may have important implications for antibody-based cancer immunotherapy. Adaptive NK cells are characterized by a heightened capacity to respond to FcγRIIIa stimulation (13), a property that has been extensively documented in the context of viral immunity but has received comparatively little attention in solid tumors. In the present study, aNK cells maintained superior functional responses following CD16 ligation despite reduced receptor expression within the TME. This functional resilience may represent an important advantage in tumors treated with therapeutic antibodies, where antibody-dependent activation constitutes a major mechanism of NK-cell-mediated immunity (35). Moreover, their enhanced ability to promote DC maturation became evident under conditions of TRZ-mediated tumor cell opsonization, suggesting that antibody-dependent activation may amplify not only their direct effector functions but also their immunoregulatory capacity. Although these findings were obtained *ex vivo* and should therefore be interpreted cautiously, they raise the possibility that aNK cells may represent a particularly relevant NK-cell subset in the context of therapeutic antibodies whose clinical efficacy relies, at least in part, on Fc-mediated immune activation.

Beyond their direct effector activity, our results suggest that aNK cells may also exert immunoregulatory functions capable of influencing the broader tumor immune response. These findings indicate that aNK cells also displayed a greater capacity to promote DC maturation in an autologous *in vitro* system using peripheral blood-derived immune cells. Although these experiments were not performed with tumor-derived DCs, they identify an additional immunoregulatory property of aNK cells that may be relevant during antibody-dependent immune responses. Given the central role of mature DC in priming naïve T cells and initiating adaptive immune responses, this observation raises the possibility that aNK cells contribute to antitumor immunity not only through direct tumor cell killing but also by facilitating the development of productive T-cell responses. DCs used in these experiments were monocyte-derived DCs obtained from healthy donors rather than DCs isolated from breast tumors. This experimental approach was selected because of the large number of cells required to establish the co-cultures. Therefore, further studies will be needed to determine whether the differential interaction between aNK cells and DC is preserved in BC patients and how it may be influenced by the TME.

Consistent with this hypothesis, increased aNK-cell infiltration was associated with a distinct immune landscape within breast tumors. Specifically, tumors enriched in aNK cells contained higher frequencies of CD8^+^ T cells co-expressing PD-1 and TIGIT, and transcriptomic analysis of the TCGA cohort revealed a positive correlation between aNK-cell and exhausted associated CD8^+^ T-cell gene signatures. These observations should be interpreted with caution, as expression of inhibitory receptors alone does not unequivocally define T-cell exhaustion and may also reflect recent or chronic antigen-driven activation (36). Nevertheless, the consistent association observed across both flow cytometric and transcriptomic analyses supports the existence of coordinated immune programs involving aNK cells and tumor-infiltrating T lymphocytes. However, this association could reflect shared recruitment or activation signals, generalized immune infiltration, or reciprocal interactions between the two populations, and further functional studies would be required to determine the underlying mechanisms. Whether aNK cells actively contribute to shaping these responses or simply accumulate in tumors remains to be determined. In support of this hypothesis, in oral squamous cell carcinoma, a marked upregulation of IFNG signatures within the tumor resident aNK cell compartment was observed in responders after ICB therapy, but not in non-responders (19). Whether aNK cells actively shape this immune landscape or preferentially accumulate in pre-existing immune-inflamed tumors remains an open question.

An additional clinically relevant observation is the association between increased adaptive NK cell associated signature infiltration and improved PFI, without relevant biological changes in OS. As a limitation we do not perform a multivariable Cox proportional-hazards analysis to determine whether the association between the aNK-associated signature and survival was independent of established prognostic factors such as breast cancer subtype, stage, age, tumor purity, or overall immune infiltration. Although these analyses do not establish causality, they suggest that adaptive NK cells may represent a favorable component of the antitumor immune infiltrate. Previous studies have demonstrated that high NK-cell infiltration is generally associated with better clinical outcomes in several malignancies (37–39) however, the contribution of individual NK-cell subsets has remained largely unexplored. Our findings extend these observations by identifying adaptive NK cells as a subset whose presence correlates with favorable prognosis, highlighting the importance of considering NK-cell heterogeneity when evaluating tumor immunity.

This study has several limitations that should be acknowledged. First, most tumors included in our cohort corresponded to luminal BC, reflecting current clinical practice, in which these patients generally undergo upfront surgery without neoadjuvant systemic treatment. Consequently, our findings may not fully capture the biology of aNK cells across other BC subtypes, particularly HER2-positive and triple-negative tumors, which more frequently receive neoadjuvant therapy before surgery. Because treatment itself profoundly modifies the tumor immune microenvironment, restricting our analysis to treatment-naïve tumors allowed us to evaluate aNK cells without the confounding effects of systemic therapy. Nevertheless, future studies including larger cohorts and additional molecular subtypes will be necessary to determine whether the phenotypic and functional properties described here are broadly conserved across breast cancer. A second limitation is that the dendritic-cell maturation assays were performed using PB-derived immune cells from healthy donors because the limited number of tumor-infiltrating immune cells precluded equivalent experiments using patient-derived tumor samples.

Collectively, our findings identify aNK cells as a functionally resilient NK-cell subset that persists within the breast TME despite phenotypic remodeling. Beyond preserving enhanced effector responses, aNK cells display increased immunoregulatory capacity and are consistently associated with favorable clinical outcome and an immune-active TME. Although the present study does not establish direct mechanistic links between these observations, it provides a conceptual framework in which aNK cells may contribute to the orchestration of antitumor immunity through mechanisms that extend beyond direct activation. Future studies aimed at defining the molecular basis of these interactions and determining whether aNK cells can be selectively expanded or therapeutically harnessed may open new opportunities to enhance antibody-based cancer immunotherapy.

Rather than acting exclusively as effector cells, adaptive NK cells may function as amplifiers of antitumor immunity by integrating antibody-dependent activation with the orchestration of downstream adaptive immune responses.

## Data Availability

The datasets presented in this study can be found in online repositories. The names of the repository/repositories and accession number(s) can be found below: https://www.ncbi.nlm.nih.gov/, The Cancer Genome Atlas (TCGA).
