# Supplementary material for: Characterization and ex vivo functional properties of NKG2C+ NK cells with adaptive features in breast tumors
Source: Front Immunol. 2026 Oct 27;17:1995578. doi: 10.3389/fimmu.2026.1995578 (PMC13748290; doi:10.3389/fimmu.2026.1995578)
Supplement: Supplementary file 1 [file Supplementaryfile1.docx]

***Supplementary Material***

# 1 Supplementary Figures and Tables

## 1.1 Supplementary Table

**Supplementary table 1.** *Fluorochrome-conjugated antibodies*

| Target molecule | Conjugated fluorophore | Clone | Brand |
| --- | --- | --- | --- |
| CD107a | PE-Cy7 | H4A3 | BD |
| CD137 | PE | 4B4-1 | BD |
| CD16 | FITC | 3G8 | BD |
| CD16 | PerCP-Cy5.5 | 3G8 | BD |
| CD25 | PE-Cy7 | M-A251 | BD |
| CD3 | APC-H7 | SK7 | BD |
| CD45 | PerCP | 2D1 | BD |
| CD56 | BV421 | NCAM16.2 | BD |
| CD56 | APC | NCAM16.2 | BD |
| CD56 | PE | NCAM16.2 | BD |
| CD69 | APC-H7 | FN50 | BD |
| CD8a | PE-Cy7 | RPA-T8 | Biolegend |
| CD80 | APC-H7 | L307.4 | BD |
| CD83 | FITC | HB15e | BD |
| CD86 | PE | 2331 (FUN-1) | BD |
| FcεRIγ | FITC | Policlonal | Millipore |
| HLA-II | PerCP-Cy5.5 | G46-6 | BD |
| IFN-γ | APC | 4S.B3 | BD |
| NKG2A | PE | 131411 | R&D systems |
| NKG2C | BV421 | 134591 | BD |
| NKp46 | PE-Cy7 | 9E2/NKp46 | BD |
| TIGIT | PE | TgMab-2 | BD |
| PD-1 | APC | MIH4 | BD |

## 1.2 Supplementary Figures

**Supplementary Figure 1.** Intratumoral aNK population selection strategy. CD45+ cells were selected first, followed by the lymphoid population based on size, cellular complexity, and single-cell events. Next, cells were selected for a probe that identifies non-viable cells, followed by CD56^+^CD3^-^ NK cells, and finally the NKG2C^-^ (cNK) and NKG2C^+^ (aNK) subpopulations. Likewise, CD8+ T lymphocytes were analyzed from the CD3+ fraction.

**Supplementary Figure 2**. Above. Experiment outline: PBMCs and cells from breast tumors were stimulated with plate-bound agonistic anti-CD16 antibody (3G8). Below: density plot showing IFNg and CD107a expression in both samples.

**Supplementary Figure 3.** CD16 engagement assay controls. **A.** Proportion of NK cells IFN-γ^+^ stimulated ON with 1 ng/ml of IL-15 (n=4). **B.** Proportion of NK cells CD107^+^ stimulated ON with 1 ng/ml of IL-15 (n=5).

**Supplementary Figure 4. A.** Histograms showing CD80, CD83 and CD86 DC maturation markers in immature dendritic cells (iDCs) in gray, mature DC (DC+medium after 5 days culture) in green and after maturation cocktail in pink. **B.** Expression of DCs maturation markers, CD83 (left), CD80 (middle) and CD86 (right), after co-culture with BT-474 cells plus TRZ, modulated by NK cells (n=5). Wald test.***p<0.001.

**Supplementary Figure 5.** Percentage of CD16 (A), CD137 (B), CD25 (C), CD69 (D) and HLA-II (E) on aNK and cNK cells stimulated with BT-474 cells and BT-474 cells opsonized with TRZ in the presence or absence of DC. Wald test *p<0.05, **p<0.01, ***p<0.001 (n=5).

**Supplementary Figure 6.** A. Pearson correlation of the proportion of aNK cells and total CD8+ T lymphocytes (left), CD8+ TIGIT+ T lymphocytes (B), and CD8+ PD-1+ T lymphocytes (C) in tumor samples.
