# Supplementary material for: Characterization and ex vivo functional properties of NKG2C+ NK cells with adaptive features in breast tumors
Source: Front Immunol. 2026 Oct 27;17:1995578. doi: 10.3389/fimmu.2026.1995578 (PMC13748290; doi:10.3389/fimmu.2026.1995578)
Supplement: Supplementary file 2 [file Supplementaryfile2.docx]

 **Table.** Post hoc pairwise comparisons between experimental conditions for each marker.** Results are from generalized linear mixed-effects models (GLMMs), with donor included as a random effect, fitted with binomial or negative binomial distributions, as appropriate. Effect estimates are presented as odds ratios (ORs) for binomial models or mean ratios for negative binomial models, together with standard errors (SEs), 95% confidence intervals (95% CIs), z-statistics, and unadjusted p-values. P-values were adjusted for multiple comparisons using the Benjamini–Hochberg false discovery rate (FDR) method. OR, odds ratio; SE, standard error; CI, confidence interval; FDR, false discovery rate.
